# Local adaptation in populations of *Mycobacterium tuberculosis* endemic to the Indian Ocean Rim

**DOI:** 10.12688/f1000research.28318.2

**Published:** 2021-03-29

**Authors:** Fabrizio Menardo, Liliana K. Rutaihwa, Michaela Zwyer, Sonia Borrell, Iñaki Comas, Emilyn Costa Conceição, Mireia Coscolla, Helen Cox, Moses Joloba, Horng-Yunn Dou, Julia Feldmann, Lukas Fenner, Janet Fyfe, Qian Gao, Darío García de Viedma, Alberto L. Garcia-Basteiro, Sebastian M. Gygli, Jerry Hella, Hellen Hiza, Levan Jugheli, Lujeko Kamwela, Midori Kato-Maeda, Qingyun Liu, Serej D. Ley, Chloe Loiseau, Surakameth Mahasirimongkol, Bijaya Malla, Prasit Palittapongarnpim, Niaina Rakotosamimanana, Voahangy Rasolofo, Miriam Reinhard, Klaus Reither, Mohamed Sasamalo, Rafael Silva Duarte, Christophe Sola, Philip Suffys, Karla Valeria Batista Lima, Dorothy Yeboah-Manu, Christian Beisel, Daniela Brites, Sebastien Gagneux

**Affiliations:** 1 Department of Medical Parasitology and Infection Biology, Swiss Tropical and Public Health Institute, Basel, Switzerland; 2 University of Basel, Basel, Switzerland; 3 Institute of Biomedicine of Valencia, Valencia, Spain; 4 Instituto de Microbiologia, Federal University of Rio de Janeiro, Rio de Janeiro, Brazil; 5 Instituto Nacional de Infectologia Evandro Chagas, Fundação Oswaldo Cruz, Rio de Janeiro, Brazil; 6 I2SysBio, University of Valencia, Valencia, Spain; 7 Institute of Infectious Diseases and Molecular Medicine, University of Cape Town, Cape Town, South Africa; 8 Department of Medical Microbiology, Makerere University, Kampala, Uganda; 9 National Institute of Infectious Diseases and Vaccinology, National Health Research Institute, Zhunan, Taiwan; 10 Institute for Social and Preventive Medicine, University of Bern, Bern, Switzerland; 11 Victorian Infectious Diseases Reference Laboratory, Melbourne, Australia; 12 Institute of Medical Microbiology, School of Basic Medical Science of Fudan University, Shanghai, China; 13 Instituto de Investigación Sanitaria Gregorio Marañón, Madrid, Spain; 14 CIBER Enfermedades Respiratorias, Madrid, Spain; 15 Servicio de Microbiología Clínica y Enfermedades Infecciosas, Hospital General Universitario Gregorio Marañón, Madrid, Spain; 16 Barcelona Institute for Global Health, Barcelona, Spain; 17 Centro de Investigação em Saúde de Manhiça, Maputo, Mozambique; 18 Ifakara Health Institute, Bagamoyo, Tanzania; 19 School of Medicine, University of California, San Francisco, USA; 20 Papua New Guinea Institute of Medical Research, Goroka, Papua New Guinea; 21 Department of Microbiology, Mahidol University, Bangkok, Thailand; 22 National Science and Technology Development Agency, Bangkok, Thailand; 23 Institut Pasteur de Madagascar, Antananarivo, Madagascar; 24 Department of Medicine, Swiss Tropical and Public Health Institute, Basel, Switzerland; 25 Université Paris-Saclay, Paris, France; 26 INSERM-Université de Paris, Paris, France; 27 Laboratório de Biologia Molecular Aplicada a Micobactérias, Fundação Oswaldo Cruz, Rio de Janeiro, Brazil; 28 Centro de Ciências Biológicas e da Saúde, Universidade do Estado do Pará, Belém, Brazil; 29 Instituto Evandro Chagas, Ananindeua, Brazil; 30 Noguchi Memorial Institute for Medical Research, University of Ghana, Accra, Ghana; 31 Department of Biosystems Science and Engineering, ETH Zürich, Basel, Switzerland

**Keywords:** Mycobacterium tuberculosis, adaptation, coevolution

## Abstract

**Background: **Lineage 1 (L1) and 3 (L3) are two lineages of the *Mycobacterium tuberculosis* complex (MTBC) causing tuberculosis (TB) in humans. L1 and L3 are prevalent around the rim of the Indian Ocean, the region that accounts for most of the world’s new TB cases. Despite their relevance for this region, L1 and L3 remain understudied.

**Methods: **We analyzed 2,938 L1 and 2,030 L3 whole genome sequences originating from 69 countries. We reconstructed the evolutionary history of these two lineages and identified genes under positive selection.

**Results: **We found a strongly asymmetric pattern of migration from South Asia toward neighboring regions, highlighting the historical role of South Asia in the dispersion of L1 and L3. Moreover, we found that several genes were under positive selection, including genes involved in virulence and resistance to antibiotics. For L1 we identified signatures of local adaptation at the *esxH* locus, a gene coding for a secreted effector that targets the human endosomal sorting complex, and is included in several vaccine candidates.

**Conclusions: **Our study highlights the importance of genetic diversity in the MTBC, and sheds new light on two of the most important MTBC lineages affecting humans.

## Introduction

The global tuberculosis (TB) epidemic is a major public health emergency, disproportionately affecting vulnerable populations and worsening existing inequalities. Although the estimated incidence and the number of fatalities have slowly decreased over time, every year ten million people develop the disease, and 1.4 million TB patients die ( WHO, 2020). Moreover, the Covid-19 pandemic will likely set back the progress toward TB eradication for several years ( Glaziou, 2020; Hogan *et al.*, 2020; McQuaid *et al.*, 2020; Saunders & Evans, 2020). TB is spread worldwide, but not all regions are equally affected: 95% of new TB cases occur in Africa and Asia, and the eight countries with the largest burden account for two thirds of the total number of cases ( WHO, 2020).

Human TB is caused by members of the *Mycobacterium tuberculosis* complex (MTBC), which includes nine phylogenetic lineages with different geographic distributions. Five of these lineages are restricted to Africa (L5, L6, L7, L8, and L9), while the remaining four (L1, L2, L3, L4) are more broadly distributed ( Chihota *et al.*, 2018; Coscollá *et al.*, 2020; Gagneux, 2018; Ngabonziza *et al.*, 2021). While L2 and L4 strains occur across the world, L1 strains are predominantly found around the rim of the Indian Ocean (East Africa, South Asia, and Southeast Asia). L3 has a distinct geographic range (East Africa, Central Asia, Western Asia, and South Asia) that overlaps partially with L1 ( Couvin *et al.*, 2019; Wiens *et al.*, 2018). While many MTBC lineages also occur in Northern Europe, North America, Australia and New Zealand, in these low-burden regions, the majority of TB cases are imported through recent migrations, and local transmission is rare ( White *et al.*, 2017).

Beyond their geographic distribution, MTBC lineages also differ in virulence, transmissibility, association with drug resistance, and the host immune responses they elicit ( Peters *et al.*, 2020). There is an increasing notion that MTBC genetic variation should be considered in the development of new antibiotics and vaccines, and when studying the epidemiology and pathogenesis of the disease ( Gagneux, 2017; Peters *et al.*, 2020; Wiens *et al.*, 2018). Yet, much of TB research to date has focused on the laboratory strain H37Rv (belonging to L4) and a few other strains belonging to L2 and L4, because of their broad geographic range, and their association with drug resistance. By contrast, L1 and L3 have largely been neglected, and only few studies have investigated the global populations of these two lineages ( Couvin *et al.*, 2019; O’Neill *et al.*, 2019). This knowledge gap is particularly severe as L1 and L3 are endemic to some of the world regions with the heaviest TB burden. For example, L1 and L3 cause the majority of TB cases in India, the country with the highest number of new TB cases in the world, and L1 is by far the most important cause of TB in the Philippines, the country with the 4 ^th^ highest global TB burden ( Netikul *et al.*, 2021; Wiens *et al.*, 2018). The aim of this study was to characterize the global population structure of L1 and L3 using large-scale population genomics, and to investigate the evolutionary history and selective forces acting on these two lineages.

## Results

**Figure 1.**
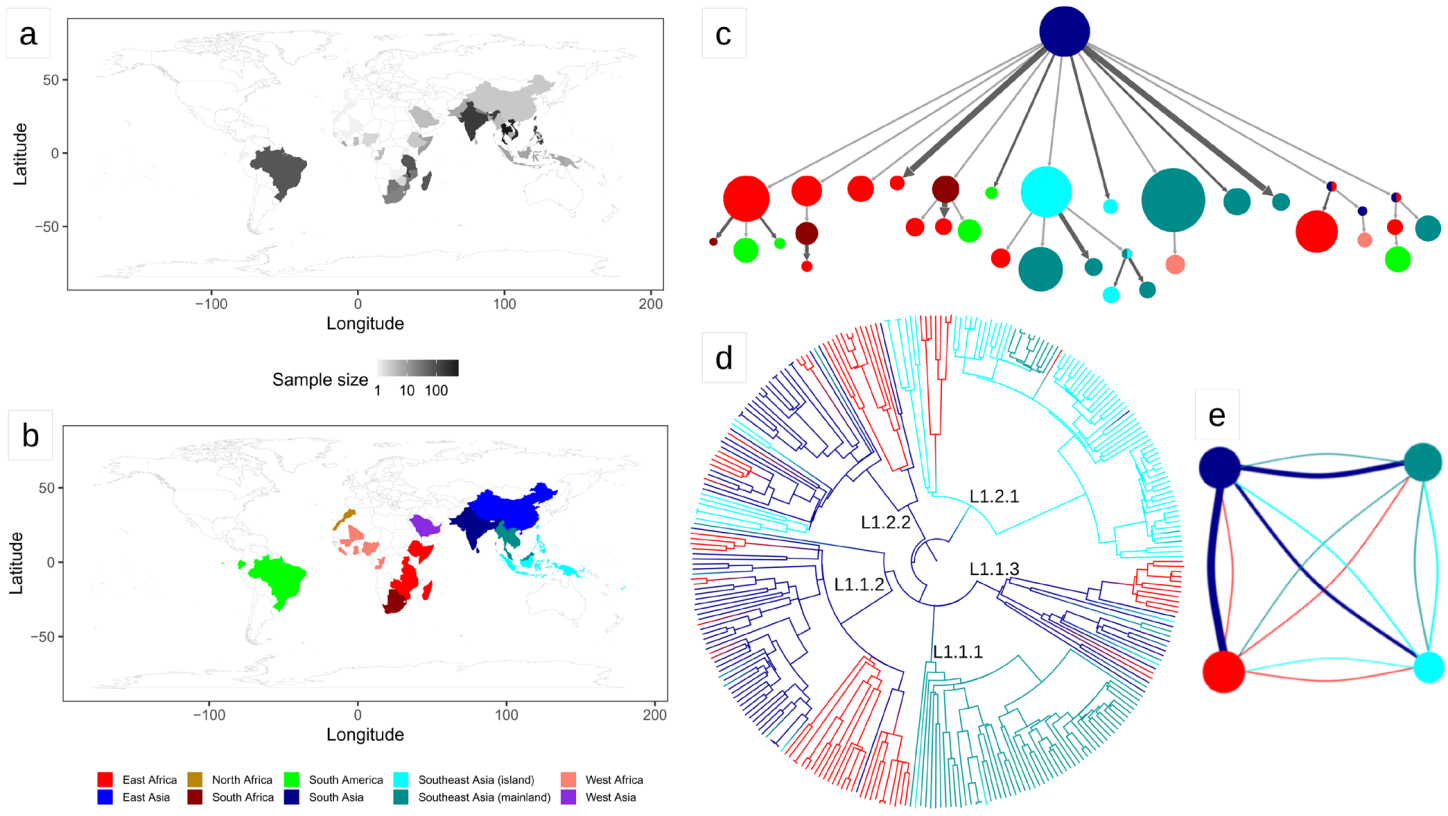
Results of the biogeography analysis: L1. **a)** Heatmap indicating the origin of the 2,061 L1 strains included in the dataset used for the biogeography analysis. **b)** The geographic regions used in the biogeography analysis of L1. **c)** Results of PASTML (compressed tree), the color code follow the legend of panel ( **b**), the size of the circles is proportional to the number of tips, and the size of the arrows is proportional to the number of times the pattern of migration was observed on the tree. This plot excluded nodes that represented less than three strains. The same plot including all nodes can be found as Extended Data File 1. **d)** Tree obtained with the Mascot analysis; the branches are colored following the legend in panel ( **b**) and represent the inferred ancestral region with the largest posterior probability. **e)** Representation of the relative effective population sizes (circles) and migration rates (lines connecting circles) estimated by Mascot. Lines representing migration rates are colored based on the region of origin (interpreted forward in time). For example, the dark blue line connecting the dark blue circle with the red circle represents the forward migration rate between South Asia and East Africa, which is the largest migration rate estimated by Mascot. The parameter estimates are reported in Extended Data Table 2.

**Figure 2.**
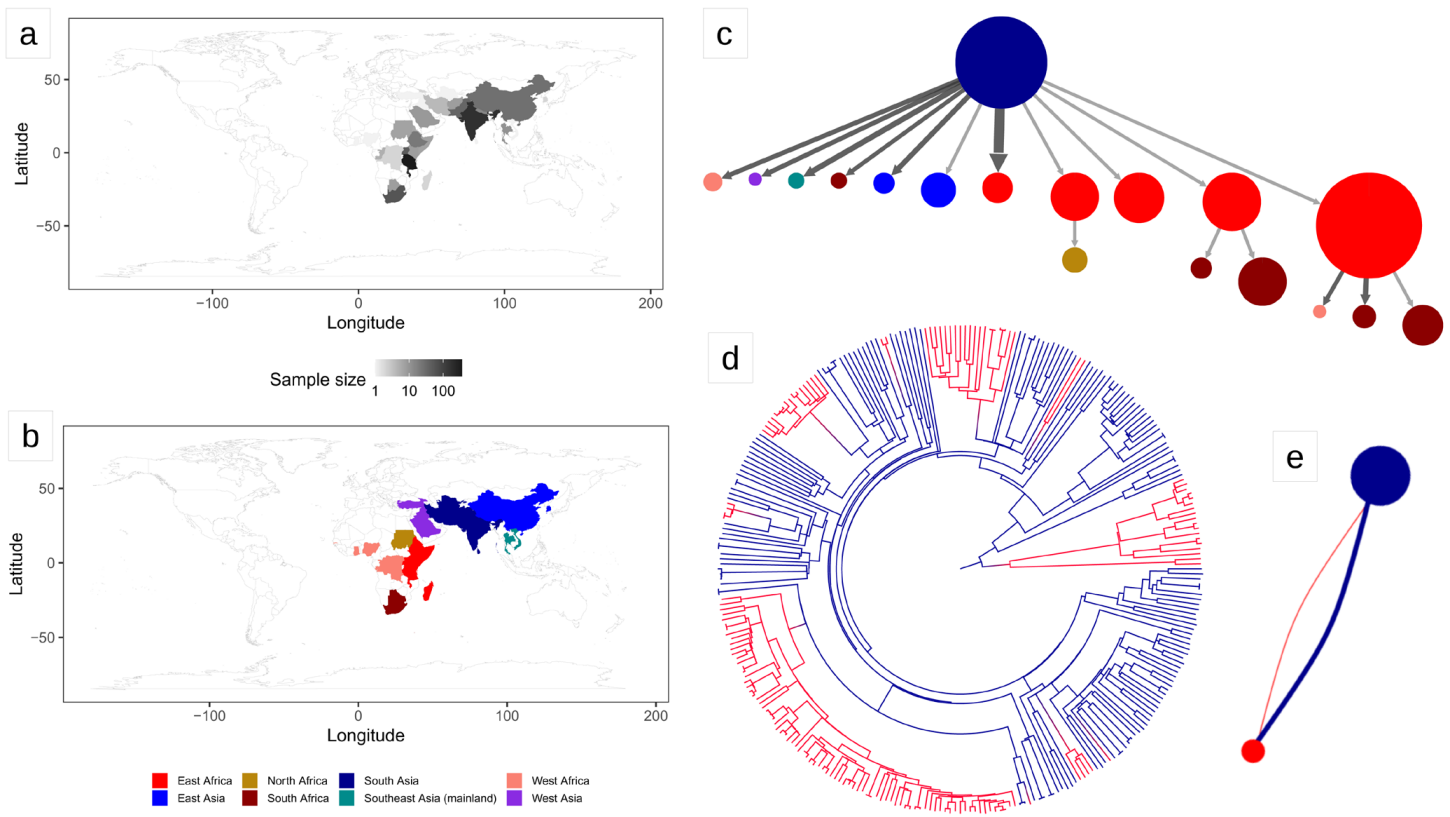
Results of the biogeography analysis: L3. **a)** Heatmap indicating the origin of 1,021 L3 strains included in the dataset used for the biogeography analysis. **b)** The geographic regions used in the biogeography analysis of L3. **c)** Results of PASTML (compressed tree), the color code follow the legend of panel (b), the size of the circles is proportional to the number of tips, and the size of the arrows is proportional to the number of times the pattern of migration was observed on the tree. This plot excluded nodes that represent less than three strains, the same plot including all nodes can be found as Extended Data File 3. **d)** Tree obtained with the Mascot analysis; the branches are colored following the legend in panel (b) and represent the inferred ancestral region with the largest posterior probability. **e)** Representation of the relative effective population sizes (circles) and migration rates (lines connecting circles) estimated by Mascot. Lines representing migration rates are colored based on the region of origin (interpreted forward in time). The effective population size of South Asia was estimated to be much larger than the one of East Africa. Additionally, the forward migration rate between South Asia and East Africa was much larger than the one in the opposite direction. The parameter estimates are reported in Extended Data Table 2.

We screened a large collection of publicly available MTBC genome sequences and selected those belonging to L1 and L3. Additionally, we newly sequenced 767 strains to cover regions that were not well represented in the public data. We applied a series of bioinformatic filters to exclude low quality sequences and mixed infections (Methods), and obtained a curated genome dataset of 4,968 strains (2,938 L1, 2,030 L3). While the phylogenetic tree of L3 showed a ladder-like topology without an evident division in sublineages, L1 comprised five clearly distinct sublineages coalescing close to the root of the tree ( *Extended data:* Figures 1 and 2).

**Figure 3.**
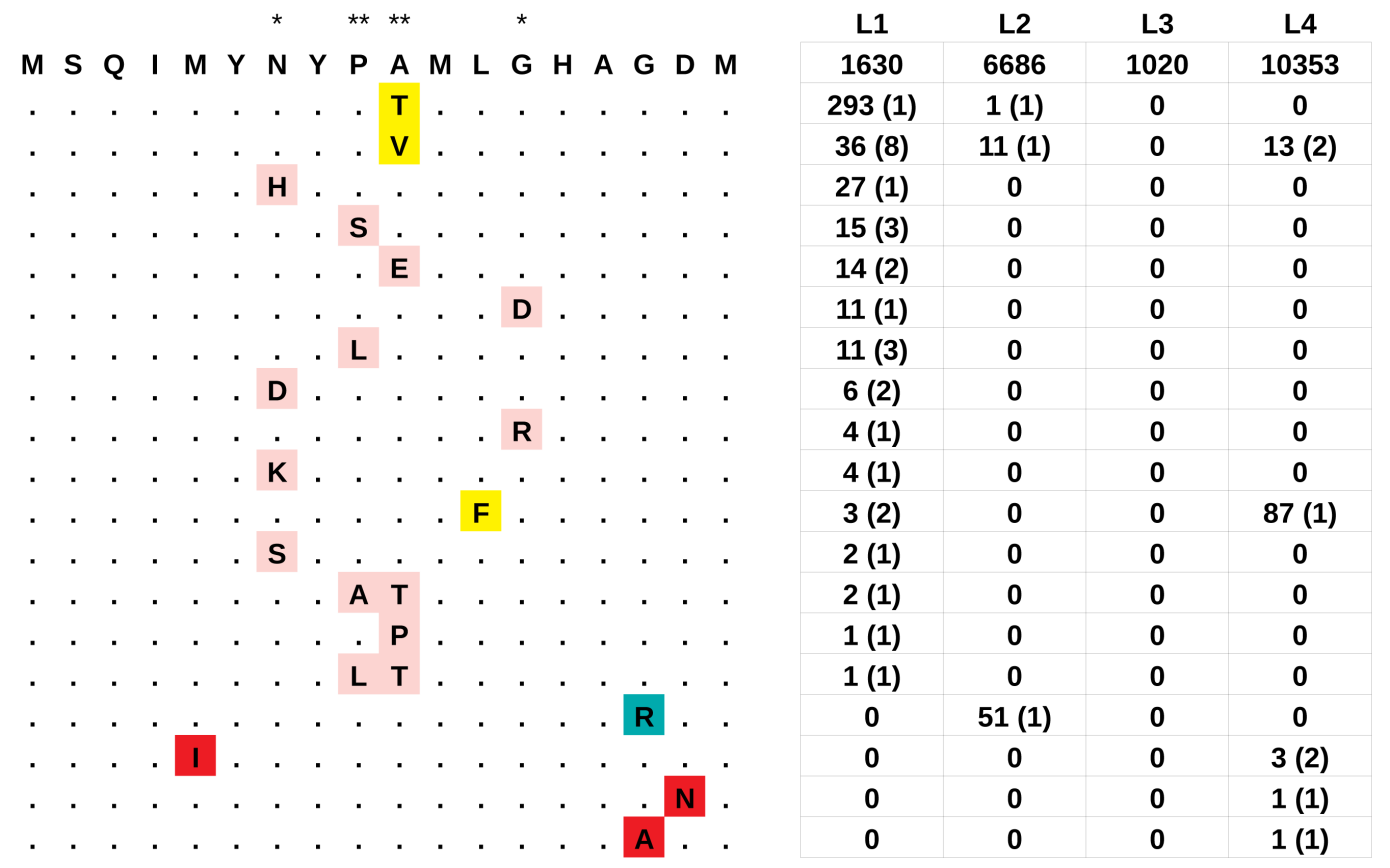
The hypervariable epitope at the N terminus of *esxH* (aa 1–18). The ancestral epitope is reported in top position, the derived haplotypes are reported below: mutations that were present in more than one lineage are highlighted in yellow, haplotypes that were found exclusively in L1, L2 or L4 are highlighted in pink, blue and red, respectively. Asterisks indicate the position inferred to be under positive selection by PAML: * posterior probability > 0.95: ** posterior probability > 0.99. The table on the right reports for each lineage the number of strains harboring the corresponding haplotype, and between parentheses, the number of independent parallel occurrences of the mutations as inferred by PAUP.

The genome sequences included in our final dataset represented 69 countries and covered the known geographic range of L1 and L3 (Methods; *Extended data:* Table 1 and Figures 3 and 4). Because we were interested in studying L1 and L3 in their endemic range, we excluded strains originating from North America, Europe, and Australia from the biogeographic analysis, as in these low-burden regions, most TB patients are recent migrants who were infected in their country of origin ( White *et al.*, 2017). We assigned strains to different geographic regions following a modified version of the United Nation geographic scheme (Methods, Figure 1 and Figure 2). Mapping the regions of origin onto the phylogenetic trees revealed that two sublineages of L1 are found almost exclusively in Southeast Asia (L1.1.1 and L1.2.1), while the others are spread over the complete geographic range of L1 ( Figure 1; *Extended Data:* Figures 5–6).

We performed a phylogeographic analysis using two alternative approaches (Mascot and PASTML), which are based on different models and inference methods ( Ishikawa *et al.*, 2019; Müller *et al.*, 2018). We found that South Asia was predicted to be the geographic range of the Most Recent Common Ancestor (MRCA) of both L1 and L3 ( *Extended Data:* Information). Most interestingly, we found a strongly asymmetric pattern of migration. For L1, PASTML identified most migration events from South Asia toward other regions, followed by further dispersion, but almost no back migration to South Asia ( Figure 1; *Extended data:* Figure 7 and Files 1 and 2). When we estimated the migration rates with Mascot, we found that the forward migration rate from South Asia toward the rest of the world were 3 to 17 times larger than the migration rates in the opposite direction, confirming the results of PASTML ( Figure 1; *Extended data*: Table 2). We found a similar scenario for L3: PASTML inferred the largest number of migrations from South Asia toward East Africa, further spread from East Africa toward neighboring regions, but essentially no migration back to South Asia ( Figure 2; *Extended data:* Files 3–4). The Mascot analysis showed that the forward migration rates from South Asia toward East Africa were 26 times larger than the migration rates in the opposite direction ( Figure 2; *Extended data:* Table 2).

We performed tip dating to estimate the age of the trees but got inconsistent results for L1, due to a lack of a reliable temporal signal ( *Extended data:* Information, Figures 8 and 9, Tables 3 and 4). However, the results of a previous study suggested a relatively fast evolutionary rate for L1 (~ 1.4×10 ^-7^ nucleotide changes per site per year; Menardo *et al.*, 2019), and that the MRCA of L1 lived around the 12 ^th^ century AD ( *Extended data:* Information). By contrast, L3 had a good temporal signal, and different methods estimated that its MRCA lived between the 2 ^nd^ and the 13 ^th^ century AD. However, the uncertainty around all of these estimates was very large ( *Extended data:* Information, Figure 8, Tables 5 and 6). Together, these results corroborate the findings of previous studies ( Bos *et al.*, 2014; Duchêne *et al.*, 2016; Menardo *et al.*, 2019). Calibrating MTBC trees that are hundreds or thousands of years old, with sequences sampled in the last few decades is notoriously challenging and subject to limitations ( Menardo *et al.*, 2019). Therefore, we refrain from any strong interpretation of the results of the molecular clock analyses of L1 and L3. We emphasize that the ages reported here are the most likely estimates supported by the available data. Additional data, or alternative methods, might result in different temporal scenarios. 

With respect to the geographical aspects, we identified several interesting instances of potential long-range dispersal. First, we found a clade of L1, composed of 11 strains sampled in five different West African countries. This was surprising because West Africa is usually not considered part of the geographic range of L1. This clade is nested within sublineage L1.1.1, which is essentially only found in mainland Southeast Asia, and the PASTML analysis inferred a direct introduction from Southeast Asia to West Africa. This introduction is unlikely to have happened before the 16 ^th^ century, when the Portuguese established the maritime route between Europe and Asia by circumnavigating Africa. We benchmarked the molecular clock of L1 and found that this scenario is indeed compatible with a clock rate equal to, or larger than 1.4×10 ^-7^ nucleotide changes per site per year, but not with lower ones ( *Extended data:* Information, Figure 10).

Second, we found that L1 was introduced to South America on at least 11 independent occasions ( Figure 1; *Extended data:* Files 1 and 2). Assuming a clock rate of 1.4×10 ^-7^, the earliest introduction was between 1620 and 1830 AD from East Africa, while subsequent introductions occurred from East Africa, South Africa, and South Asia. These results support the hypothesis that L1 was first introduced to Brazil through the slave trade from East Africa ( Allen, 2013; Conceição *et al.*, 2019), although, due to the lack of samples from other South American countries, we cannot exclude a more complex evolutionary history of L1 in this continent. Interestingly, this is in contrast with L5 and L6, which are endemic to West Africa, but did not establish themselves firmly in South America ( De Jong *et al.*, 2010; Rabahi *et al.*, 2020).

Third, similar to the West African clade of L1.1.1, we found an East African clade embedded within sublineage L1.2.1, which otherwise is found almost exclusively in Southeast Asia. This East African clade is composed of 11 strains from five countries, and its sister clade is found in East Timor and Papua New Guinea. We inferred a direct migration from the islands of Southeast Asia to East Africa that occurred between the 13 ^th^ and the 16 ^th^ century AD (assuming a clock rate of 1.4×10 ^-7^). This would be compatible with early Portuguese expeditions, which reached East Timor and Papua New Guinea in the early 16 ^th^ century. An alternative explanation could be the Austronesian expansion. Austronesians are thought to have reached the Comoros islands and Madagascar between the 9 ^th^ and the 13 ^th^ century AD, possibly through direct navigation from Southeast Asian islands. Malagasy speak an Austronesian language, and Austronesian genetic signatures are found in human populations in the Comoros, Madagascar, and to a small extent also in the Horn of Africa ( Blench, 2010; Boivin *et al.*, 2013; Brucato *et al.*, 2016; Brucato *et al.*, 2018; Brucato *et al.*, 2019; Pierron *et al.*, 2014).

L1 and L3 coexist in many regions around the Indian Ocean. Yet, in their evolutionary history, these lineages colonized areas occupied by different human populations. Human genetic variation has been shown to influence the susceptibility to TB ( Qu *et al.*, 2011). Most notably, human leukocyte antigen (HLA) genes play a crucial role in the activation of the immune responses to the MTBC by exposing bacterial peptides (epitopes) to the surface of an infected cell, where they can be recognized by T cells. HLA genes are extremely polymorphic in human populations, and several alleles of different HLA genes are associated with TB susceptibility in different populations ( Brahmajothi *et al.*, 1991; Salie *et al.*, 2014; Sveinbjornsson *et al.*, 2016; Vejbaesya *et al.*, 2002; Yuliwulandari *et al.*, 2010).

Previous studies have shown that T cell epitopes are hyper-conserved in the MTBC, suggesting that immune escape does not provide an advantage and that, contrary to other pathogens, the MTBC needs to be recognized by the immune system and to cause disease in order to transmit ( Comas *et al.*, 2010; Coscolla *et al.*, 2015). Our large dataset of L1 and L3 genome sequences from different geographic regions provided an opportunity to scan for lineage- and/or region-specific signatures of selection at T cell epitopes in L1 and L3.

We reconstructed the mutational history of T cell epitopes in L1 and L3, and found that 51% of all epitopes were variable (at the amino acid level) in at least one L1 strain, while only 20% were variable in at least one L3 strain ( *Extended data:* Table 7). However, this difference can be explained by the different size and diversity of the two datasets (2,061 genome sequences with 136,023 variable sites for L1; 1,021 genome sequences with 36,316 variable sites for L3). The epitope that accumulated most mutations was located at the N terminus of *esxH* ( *Rv0288*). This peptide is a T cell epitope for both classes, MHCI and MHCII; it is also a B cell epitope and was previously identified as one of the few T cell epitopes that were not hyper-conserved ( Comas *et al.*, 2010). We found 15 derived haplotypes (at the amino acid level), generated by 28 independent replacements at five positions in a peptide of seven amino acids ( Figure 3). By contrast, the second most variable epitope accumulated only seven amino acid changes ( *Extended data*: Table 7). Interestingly, we did not find this signature in L3, as all strains carried the ancestral haplotype at the N-terminal *esxH* epitope. Moreover, when we extended the analysis to two large genomic datasets of L2 and L4 strains, we found a much weaker signal: while 21% of L1 strains carried a derived haplotype for this epitope, only 1% of L2 and L4 strains, and no L3 strain had a derived haplotype. Despite analyzing datasets with more strains (6,752 and 10,466 for L2 and L4, respectively), and more polymorphic positions (140,309 and 277,648) compared to L1, we found only three amino acid replacements at the N-terminal epitope of *esxH* in L2, and seven in L4 ( Figure 3). The most frequently mutated position was the tenth amino acid, where we found 12 independent replacements in L1, and two in L2 and L4 ( Figure 3). The amino acid replacement A10V occurred eight times in parallel in L1, once in L2 and twice in L4. The most abundant derived haplotype was caused by a different amino acid replacement at position ten (A10T), which occurred once in L1 and once in L2 ( Figure 3). Overall, the replacements in all lineages occurred in eight residues in a peptide of 13 amino acids ( Figure 3).

We evaluated the robustness of these results by formally testing for positive selection on the complete sequence of *esxH* using PAML ( Yang, 2007). We found that *esxH* was indeed under positive selection in L1 (p-value = 0.00004) but not in the other lineages (p-values = 0.39, 1.00 and 0.65 for L2, L3 and L4, respectively). PAML identified four codons that have been under positive selection in L1 (posterior probability > 0.95), all of them within the N-terminal epitope (codons 7, 9, 10 and 13; Figure 3). Codon 76, which is part of a different T cell epitope, had a posterior probability > 0.9, mutated three times in parallel and was possibly also under positive selection.

Our results further revealed that the derived haplotypes of the N-terminal *esxH* epitope were not distributed randomly across the geographic range of L1. Twenty-two of the 28 (79%) amino acid replacements occurred in sublineages L1.1.1 and L1.2.1, which are almost exclusively present in Southeast Asia ( *Extended data:* Figure 11). We constructed a statistical test of association similar to phyC ( Farhat *et al.*, 2013) to determine whether replacements in the hypervariable *esxH* epitope were significantly associated with a particular geographic region (Methods). We found that South African strains were less likely to harbor a derived haplotype in the N-terminal *esxH* epitope than expected by chance (empirical p-value = 0.013; Table 1). While East African L1 strains were not associated with the derived haplotypes (empirical p-value = 0.276), we noticed important differences between countries: of the 29 East African strains harboring a derived haplotype, 28 were sampled in Madagascar. When we excluded Madagascar, we found that East Africa had a strong negative association with the derived haplotypes (i.e. East African strains harbored less derived haplotypes than expected by chance; empirical p-value = 0.0004). We then tested the most frequently replaced position (position ten) alone, and again found that East African strains were negatively associated with the derived haplotypes, with and without excluding Malagasy strains (empirical p-values = 0.0176 and 0.034, respectively). Finally, we tested the derived haplotype caused by the most frequent amino acid replacement (A10V; 8 parallel occurrences). Again, we found a negative association with East African strains (empirical p-values = 0.046 and 0.079, respectively including and excluding Malagasy strains; Table 1).

**Table 1.** Results of the test of association between haplotypes of the N-terminal *esxH* epitope and the geographic region of origin.

Region ^1^	Strains ^2^	Association ^3^	*esxH* (A10V) ^4^		*esxH* (A10X) ^5^		*esxH* (N terminus) ^6^	
			Strains	p-value	Strains	p-value	Strains	p-value
South Asia	219	**+**	1	0.4852	6	0.385	17	0.453
Southeast Asia (islands)	235	**+**	10	0.154	156	0.081	171	0.174
Southeast Asia (mainland)	982	**+**	25	0.200	183	0.095	210	0.196
East Africa	466	**-**	0	0.046	1	0.034	29	0.276
East Africa (no Madagascar)	391	**-**	0	0.070	0	0.018	1	0.0004
South Africa	49	**-**	0	0.305	0	0.165	0	0.013
South America	77	**-**	0	0.496	0	0.352	0	0.095

^1^ We included only regions for which the sample size was 25 or more.

^2^ Total number of L1 strains from each region.

^3^ All p-values are one-tailed empirical p-values. This column indicates the direction of the association: + we tested for positive association between the derived haplotype(s) and the geographic region; - we tested for negative association between derived haplotype(s) and the geographic region.

^4^ Number of strains with the derived haplotype caused by the replacement A10V, and results of the test of association.

^5^ Number of strains with the derived haplotypes caused by any replacement at position 10 (A10X), and results of the test of association.

^6^ Number of strains with the derived haplotypes caused by any non-synonymous mutations occurred in the first 18 codons of *esxH*, and results of the test of association.

We hypothesized that the accumulation of missense mutations at the N-terminal epitope of *esxH* was due to immune escape. Therefore, we performed *in silico* prediction of the binding affinity of the ancestral haplotype and of the two most frequent derived haplotypes (caused by the amino acid replacements A10V and A10T) with different HLA-A, HLA-B and HLA-DRB1 alleles (Methods). We performed this analysis for:

1) Alleles found at high frequency (> 10%) in South- and Southeast Asia, but not in East Africa.

2) Alleles found at high frequency (> 10%) in East Africa, but not in South- and Southeast Asia.

3) Alleles found at high frequency (> 10%) in both regions.

However, we found no differences in the predicted binding affinities between alleles with different geographic distributions ( *Extended data:* Table 8).

While *esxH* was the most striking example of a selective pressure specific for one lineage, our analysis suggests that it was not an isolated case. We performed a genome-wide scan for selection with PAML, and identified 17 genes under positive selection, of which five in common between L1 and L3 ( *Extended data:* Table 9). We found two genes coding for transmembrane proteins, members of the Esx-1 secretion system, which were under positive selection in L1 ( *eccB1* and *eccCa1*, Bonferroni corrected p-values = 0.02 and 0.03), and several genes involved in antibiotic resistance that were under positive selection in both lineages ( *Extended data:* Information). We further characterized the profile of drug resistance mutations of L1 and L3, and found that L1 strains harbored a greater proportion of *inhA* promoter mutations (conferring resistance to isoniazid) compared to L3 strains, confirming previous findings ( Fenner *et al.*, 2012; *Extended data:* Information, Figure 12, Table 10). 

## Discussion

Our results highlight the central role of South Asia in the dispersion of L1 and L3. First, we confirmed that the two lineages probably expanded from South Asia ( O’Neill *et al.*, 2019). Second, contrary to previous studies that assumed symmetric migration rates between regions ( O’Neill *et al.*, 2019), we found that these migrations occurred mostly in one direction. South Asia was a source of migrant strains that fueled the epidemics in other regions, especially in East Africa. Historically, a network of maritime trade, which followed the seasonality of the Monsoons, connected East Africa and South Asia. It is unclear how this could have promoted the spread of TB in one direction, but not in the other. A possible explanation is that strains originating in South Asia were more efficient in transmitting in East Africa, compared to East African strains that migrated to South Asia.

Another difference compared to previous studies is the temporal framework for the evolutionary history of L1. O’Neill *et al.* (2019) estimated that the MRCA of L1 lived in the 4 ^th^ century BC and that the migration rates decreased after the 7 ^th^ century AD. Our results indicate that the MRCA of L1 did not exist before the 12 ^th^ century AD. This discrepancy is due to different assumptions about the clock rates of L1, but none of the two hypotheses can be excluded with the available data. Nonetheless, our discovery of a West African clade that likely originated through a direct introduction from Southeast Asia supports a faster clock rate of L1, and therefore a more recent MRCA ( *Extended data*: Information). As we already mentioned, tip-dating analyses of MTBC trees with roots that are several hundreds of years old is extremely challenging, and the results of such analyses should be taken with caution ( Menardo *et al.*, 2019). Moreover, all MTBC molecular clock studies so far assumed that evolutionary rate estimates do not depend on the age of the calibration points ( Ho *et al.*, 2005). There are some indications that the effect of time dependency in MTBC datasets is negligible ( Pepperell *et al.*, 2013; Menardo *et al.*, 2019). However, this assumption has not yet been thoroughly tested. Keeping in mind these limitations, and tentatively accepting the results of the molecular clock analyses, we can compare the biogeography of L1 and L3 with the results obtained for other MTBC lineages. The expansion of L1 and L3 to East Africa, and of L1 to Southeast Asia, seems to have started before the 16 ^th^ century, earlier compared to the introduction of L2 and L4 to Africa, but similar to what inferred for the migration of L4 to Southeast Asia from Europe ( Brynildsrud *et al.*, 2018; Rutaihwa *et al.*, 2019). After the onset of European explorations in the 15 ^th^ century, L1 was introduced to West Africa and South America. This is in line with what observed for L4, and for the European clonal complexes of *M. bovis*, which expanded to the Americas and to Oceania after the 15 ^th^ century ( Brynildsrud *et al.*, 2018; Loiseau *et al.*, 2020; Mulholland *et al.*, 2019).

We found evidence for a strong selective pressure acting on the N terminus of *esxH* in L1. In contrast, this selective pressure seems to be much reduced, if not absent, in the “modern” lineages (L2, L3 and L4). It is known that L1 strains interact differently with the infected hosts compared to other lineages. For example, L1 strains show a lower virulence in animal models ( Bottai *et al.*, 2020), transmit less efficiently in some clinical settings, and infect older patients ( Holt *et al.*, 2018). It has been shown recently that the increased virulence of the so-called “modern” MTBC strains is due to the loss of the genomic region TbD1, which remains present in L1 ( Bottai *et al.*, 2020). However, it was also reported that in some populations, L1 was associated with higher patient mortality ( Smittipat *et al.*, 2019). Given these differences with the “modern” lineages, it is likely that L1 is subject to different selective pressures, and it is possible that the greater selective pressure on *esxH* was caused by some epistatic effect specific to L1.

The signatures of selection at the N-terminal epitope of *esxH* were associated with strains sampled in South- and Southeast Asia, and were almost completely absent in East Africa (excluding Madagascar). Region-specific signatures of positive selection are a hallmark of local adaptation; in this case, adaptation of L1 strains to human hosts with South- and Southeast Asian genotypes. This corroborates previous studies reporting that in Taiwan, L1 is associated with indigenous populations with Austronesian ancestry (reviewed in Dou *et al.*, 2015). This hypothesis is also supported by the L1 population in Madagascar. Madagascar is geographically linked to East Africa, however, Malagasies are genetically distinct from Africans, as they have mixed African and Southeast Asian ancestry due to the Austronesian colonization of Madagascar ( Brucato *et al.*, 2016). Madagascar was the region with the second highest prevalence of derived haplotypes in the N-terminal epitope of *esxH* after Southeast Asia (islands), 37.3% and 72.8% respectively, opposed to 0.3% (one single strain) in the rest of East Africa.

Although all the codons under positive selection in *esxH* were contained in one single T cell epitope, the selective pressure acting on *esxH* could be due to some other factor, and not to the binding of the epitope by the MHC, or to its recognition by T cells. A possible mechanism was suggested by experiments in a mouse model, showing that the N-terminal epitope of EsxH generates an immunodominant CD8 T cells response. The amino acid replacement A10T (which we found to be the most prevalent among the derived haplotypes of the N-terminal EsxH epitope) did not change MHC binding or T cell recognition, but it accelerated the degradation of the epitope, disrupting the immunodominant response ( Sutiwisesak *et al.*, 2020; Woodworth *et al.*, 2008). 

An additional driver of selection could be the effector function of EsxH. EsxH is a small effector secreted by the Esx-3 secretion system as dimer with EsxG ( Ilghari *et al.*, 2011). Within the host macrophages, the dimer EsxH-EsxG targets Hrs ** (a component of the human endosomal sorting complex), impairing trafficking, and hindering phagosomal maturation and antigen presentation, thus contributing to the survival of the pathogen ( Mehra *et al.*, 2013; Mittal *et al.*, 2018; Portal-Celhay *et al.*, 2016). The observed signatures of selection could be due to the adaptation of L1 strains to human genotypes of *hrs* prevalent in South- and Southeast Asia. A similar signature of selection was observed in another Esx effector (EsxW), in MTBC strains belonging to L2 ( Holt *et al.*, 2018). Holt and colleagues found evidence for parallel evolution at one residue in the N-terminal loop of EsxW, outside the region covered by known epitopes, suggesting that the selective pressure on EsxW was not due to antigen recognition.

The sampling effort in this study was considerable, and it provided a more complete picture compared to previous studies. Nevertheless, the sampling was not population-based, and for some regions the coverage was scarce (e.g. the Arabian Peninsula). Because of these limitations, our biogeographical analyses were limited to the subcontinental level. This approach revealed the global population structure and the main macroscopic patterns of diversity and migration of L1 and L3. However, MTBC populations are diverse also within subcontinental regions. For example, the MTBC population in Southern India is dominated by L1, while the most prevalent lineage in the North is L3 ( Couvin *et al.*, 2019). To investigate fine-scale processes in greater detail, including local adaptation, large population-based studies will be necessary.

In conclusion, the results presented here improve our knowledge about the TB epidemic around the Indian Ocean. A better understanding of the evolutionary dynamics of different MTBC populations might inform the development of control strategies for different regions. For example, *esxH* is part of several new vaccine candidates ( Abel *et al.*, 2010; Hoang *et al.*, 2013; Radošević *et al.*, 2007), and at least one of these, H4:IC31, is under clinical development in South Africa ( Bekker *et al.*, 2020; Nemes *et al.*, 2018). In the light of our findings, and to develop a globally effective vaccine, it would be important to know if the results of the clinical trials in South Africa can be replicated in Southeast Asia, where there is a high prevalence of derived *esxH* haplotypes in the circulating MTBC populations.

## Methods

### Strain cultures, DNA extraction and genome sequencing

MTBC isolates previously identified as L1 and L3, either by SNP-typing or spoligotyping, were grown in Middlebrook 7H9 liquid medium supplemented with ADC and incubated at 37°C. Purified genomic DNA was obtained from cultures using a CTAB extraction method ( Belisle & Sonnenberg, 1998). Whole genome sequencing was performed on libraries prepared from purified genomic DNA using Illumina Nextera ® XT library and NEBNext ® Ultra TM II FS DNA Library Prep Kits. Sequencing was performed using the Illumina HiSeq 2500 or NextSeq 500 paired-end technology.

The sequence data generated by this study has been deposited on SRA under the accession number PRJNA670836.

### Bioinformatic pipeline

We screened a large collection of publicly available whole genome sequences (Illumina) of MTBC strains belonging to L1 and L3, using the diagnostic SNPs described in Steiner *et al.* (2014). To cover geographic regions that were under-represented by this dataset, we additionally sequenced the genomes of 767 clinical strains (360 L1, and 407 L3).

For all samples, Illumina reads were trimmed with Trimmomatic v0.33 (SLIDINGWINDOW: 5:20,ILLUMINACLIP:{adapter}:2:30:10) ( Bolger *et al.*, 2014). Reads shorter than 20 bp were excluded from the downstream analysis. Overlapping paired-end reads were then merged with SeqPrep (overlap size = 15; https://github.com/jstjohn/SeqPrep). The resulting reads were mapped to the reconstructed MTBC ancestral sequence ( Comas *et al.*, 2013) with BWA v0.7.12 (mem algorithm; Li & Durbin, 2010). Duplicated reads were marked by the MarkDuplicates module of Picard v 2.1.1 ( https://github.com/broadinstitute/picard). The RealignerTargetCreator and IndelRealigner modules of GATK v.3.4.0 ( McKenna *et al.*, 2010) were used to perform local realignment of reads around Indels. Reads with alignment score lower than (0.93*read_length)-(read_length*4*0.07)) were excluded: this corresponds to more than 7 miss-matches per 100 bp.

SNPs were called with Samtools v1.2 mpileup ( Li *et al.*, 2009) and VarScan v2.4.1 ( Koboldt *et al.*, 2012) using the following thresholds: minimum mapping quality of 20, minimum base quality at a position of 20, minimum read depth at a position of 7X, minimum percentage of reads supporting the call 90%. SNPs in previously defined repetitive regions were excluded (i.e. PPE and PE-PGRS genes, phages, insertion sequences and repeats longer than 50 bp) as described before ( Brites *et al.*, 2018).

We applied the following filters: genomes were excluded if they had 1) an average coverage <15x, 2) more than 50% of their SNPs excluded due to the strand bias filter, 3) more than 50% (or more than 1,000 in absolute number) of their SNPs having a percentage of reads supporting the call between 10% and 90%, 4) contained single nucleotide polymorphisms diagnostic for different MTBC lineages ( Steiner *et al.*, 2014), as this indicated that a mix of genomes was sequenced, 5) had more than 5,000 SNPs of difference compared to the reconstructed ancestral genome of the MTBC ( Comas *et al.*, 2013). Additionally, when multiple strains were sampled from the same patient, we retained only one. We further excluded all strains that had less SNPs than (average - (3 * standard deviation)) of the respective lineage (calculated after all previous filtering steps). We built SNP alignments for L1 and L3 separately, including only variable positions with less than 10% of missing data, and finally, we excluded all genomes with more than 10% of missing data in the alignment of the respective lineage. After all filtering steps, we were able to retrieve 4,968 strains with high quality genome sequences for further analyses (2,938 L1, 2,030 L3; *Extended data:* Table 1).

### Analysis of sublineages

We used the curated datasets and inferred phylogenetic trees based on all polymorphic positions (excluding the ones in repetitive regions, see above) with raxml 8.2.11 ( Stamatakis, 2014; -m GTRCAT and -V options). We then identified sublineages following the classification (and using the diagnostic SNPs) of Coll *et al.* (2014).

### Molecular clock analyses with LSD

We selected all strains for which the year of sampling was known (2,499 strains, 1,672 L1, 827 L3). For both lineages, we built SNP alignments including only variable positions with less than 10% of missing data. We inferred phylogenetic trees with RAxML 8.2.11 ( Stamatakis, 2014), using the GTR model (-m GTRCAT -V options). Since the alignments contain only variable positions, we rescaled the branch lengths of the trees: rescaled_branch_length = ((branch_length * alignment_lengths) / (alignment_length + invariant_sites)). We rooted the trees using the genome sequence of a L2 strain as outgroup (accession number SAMEA4441446).

We used the least square method implemented in LSD v0.3-beta ( To *et al.*, 2016) to estimate the molecular clock rate with the QPD algorithm and calculating the confidence interval (options -f 100 and -s). We also performed a date randomization test by randomly reassigning the sampling dates among taxa 100 times and estimating the clock rate from the randomized and observed datasets. All LSD analyses were performed on the two lineages individually (L1 and L3), and on the five sublineages of L1.

### Molecular clock analyses with Beast

Bayesian molecular clock analyses are computationally demanding and they would be impossible to apply onto the complete datasets. Therefore, we sub-sampled the L1 and L3 datasets used for the LSD analysis to 400 genomes with two different strategies: 1) random subsampling 2) random subsampling keeping at least 25 genomes for each year of sampling (where possible; “weighted subsampling”). For this second subsampling strategy, we used Treemmer v0.3 ( Menardo *et al.*, 2018) with options -X 400, -pr, -lm, and -mc 25. For both subsampling schemes, we generated three subsets of the original datasets, resulting in six subsets for each lineage. We assembled SNP alignments including only variable positions with less than 10% of missing data, and used jModelTest 2.1.10 v20160303 ( Darriba *et al.*, 2012) to identify the best fitting nucleotide substitution model (according to the Akaike information criterion) among 11 possible schemes including unequal nucleotide frequencies (total models = 22, options -s 11 and -f).

We performed Bayesian inference with Beast 2.5 ( Bouckaert *et al.*, 2019). We corrected the xml files to specify the number of invariant sites as indicated here: https://groups.google.com/forum/#!topic/beast-users/QfBHMOqImFE, and used the tip sampling year as calibration. We assumed the best fitting nucleotide substitution model as identified by jModelTest, a relaxed lognormal clock model ( Drummond *et al.*, 2006) and an exponential population size coalescent prior. We chose a 1/x prior for the population size [0–109], a 1/x prior for the mean of the lognormal distribution of the clock rate [10−10–10−5], a normal(0,1) prior for the standard deviation of the lognormal distribution of the clock rate [0 –infinity]. For the exponential growth rate prior, we used the standard Laplace distribution [-infinity–infinity]. For all datasets, we ran at least two runs, we used Tracer 1.7.1 ( Rambaut *et al.*, 2018) to identify and exclude the burn-in, to evaluate convergence among runs and to calculate the estimated sample size. We stopped the runs when at least two chains reached convergence, and the ESS of the posterior and of all parameters were larger than 200.

Since we detected a strong temporal signal only for L3, we performed a set of additional analyses of the subsets of L3. We repeated the Beast analysis with an extended Bayesian Skyline Plot (BSP) prior instead of the exponential growth prior, and performed a nested sampling analysis ( Russel *et al.*, 2019) to identify which of these two models (exponential growth and extended BSP) fitted the data best. The nested sampling was run with chainLength = 20000, particleCount= 4, and subChainLength = 10000.

All xml input files are available as Supplementary Files in *Extended data.*

### Datasets for biogeography analyses

For the biogeography analysis, we considered only genome sequences obtained from strains for which the locality of sampling was known. When the country of sampling did not correspond to the country of origin of the patient (or was unknown), we considered as sampling locality the country of origin of the patient (this affected 187 strains, 121 L1 and 66 L3). Furthermore, similarly to other studies ( O’Neill *et al.*, 2019), we excluded all genomes that were sampled from Europe, North America and Australia, as most contemporary infections in these regions affect recent migrants. The final dataset for the biogeography analyses comprised 3,082 strains (2,061 L1 and 1,021 L3). We assigned the different isolates to different subcontinental geographic regions according to sampling locality. To do this, we followed a modified version of the United Nations geographic scheme ( https://unstats.un.org/unsd/methodology/m49/; *Extended data:* Table 1 and Figures 1–3).

### Phylogeography analysis with PASTML

For both lineages, we built SNP alignments including only variable positions with less than 10% of missing data, and all strains with known sampling locality (excluding strains from North America, Europe and Australia; see above, 2,061 L1 genomes and 1,021 L3 genomes). We inferred phylogenetic trees with raxml 8.2.11 ( Stamatakis, 2014), using the GTR model (-m GTRCAT -V options). Since the alignments contain only variable positions, we re-scaled the branch lengths of the trees: rescaled_branch_length = ((branch_length * alignment_lengths) / (alignment_length + invariant_sites)).

Since PASTML needs a time tree as input, we calibrated the phylogenies with LSD, assuming a clock rate of 1.4×10 ^-7^ for L1, and 9×10 ^-8^ for L3. In this analysis, genomes for which the sampling date was not known were assumed to have been sampled between 1995 and 2018, which is the period in which all strains with known date of isolation were sampled. Importantly, using different clock rates for this analysis would only change the time scale of the trees, but not the reconstruction of the ancestral characters.

We assigned to each strain the subcontinental geographic region of origin as character, and used PASTML ( Ishikawa *et al.*, 2019) to reconstruct the ancestral geographical ranges and their changes along the trees of L1 and L3. We used the MPPA as prediction method (standard settings) and added the character predicted by the joint reconstruction even if it was not selected by the Brier score (option -forced_joint). Additionally, we repeated the PASTML analysis for the sublineages of L1 individually.

### Phylogeography analysis with Mascot

As a complementary method to reconstruct the ancestral range and the migration pattern of different populations, we used the Beast package Mascot ( Müller *et al.*, 2018). We assumed that strains sampled in the different subcontinental regions represent distinct subpopulations, and we considered only populations for which we had at least 75 genome sequences: four populations for L1 (East Africa, South Asia, Southeast Asia (mainland) and Southeast Asia (islands)), and two populations for L3 (East Africa and South Asia). For computational reasons, we subsampled the two datasets (L1 and L3) to ~300 strains. We sampled an equal number of strains from each geographic region (where possible), and within regions, we randomly sampled an equal number of strains from each country (where possible). This sub-sampling scheme resulted in two subsets of 303 and 300 strains for L1 and L3, respectively.

We assembled SNP alignments including only variable positions with less than 10% of missing data, and used jModelTest 2.1.10 v20160303 ( Darriba *et al.*, 2012) to identify the best fitting nucleotide substitution model as described above.

We performed Bayesian inference with Beast2.5 ( Bouckaert *et al.*, 2019). We corrected the xml files to specify the number of invariant sites as indicated here: https://groups.google.com/forum/#!topic/beast-users/QfBHMOqImFE. We assumed a lognormal uncorrelated clock and we fixed the mean of the lognormal distribution of the clock rate to 1.4×10 ^-7^ (L1) and 9×10 ^-8^ (L3). We assigned the tip sampling years to the different strains, and when the sampling time was unknown, we assumed a uniform prior from 1995 to 2018 (similarly to what done in the PASTML analysis). We further assumed the best fitting nucleotide substitution model as identified by jModelTest, a gamma prior for the standard deviation of the lognormal distribution of the clock rate [0 –infinity], and a lognormal prior for the population size with standard deviation = 0.2, and mean estimated in real space. Finally, we used an exponential distribution with mean = 10 ^-4 ^as prior for the migration rates. For each analysis, we ran at least two runs. We used Tracer 1.7.1 ( Rambaut *et al.*, 2018) to identify and exclude the burn-in, to evaluate convergence among runs, and to calculate the estimated sample size. We stopped the runs when at least two chains reached convergence, and the ESS of the posterior, prior and of the parameters of interest (population sizes and migration rates) were larger than 200.

The xml input files are available as Supplementary Files in *Extended data.*

### L2 and L4 datasets

For the analysis of *esxH*, we wanted to compare the results obtained for L1 and L3 with the other two major human-adapted MTBC lineages L2 and L4. Therefore, we compiled two datasets of publicly available genome sequences for these two lineages. We applied the same bioinformatic pipeline described above: genomes were excluded if they had 1) an average coverage <15x, 2) more than 50% (or more than 1,000 in absolute number) of their SNPs having a percentage of reads supporting the call between 10% and 90%, or 3) contained single nucleotide polymorphisms diagnostic for different MTBC lineages ( Steiner *et al.*, 2014), as this could indicate mixed infection. The final dataset consisted of 6,752 L2 genome sequences (with 140,309 polymorphic positions) and 10,466 L4 genome sequences (with 277,648 polymorphic positions; *Extended data:* Table 1). We reconstructed the phylogenetic tree of L2 with raxml 8.2.11 ( Stamatakis, 2014) as described above. Due to the large size of the dataset, we used FastTree ( Price *et al.*, 2010) with options -nt -nocat -nosupport to reconstruct the phylogenetic tree of L4.

### Epitopes analysis

We downloaded the amino acid sequence of all MTBC epitopes described for *Homo sapiens* from the immune epitope database ( https://www.iedb.org/; downloaded on the 03.08.2020). We considered separately MHCI epitopes and MHCII epitopes, and we analyzed overlapping epitopes individually. We mapped the epitope sequences onto the H37Rv genome (GCF_000195955.2) using tblastn and excluded sequences mapping equally well to multiple loci in the H37Rv genome. Additionally, we retained only epitopes that mapped with two mismatches or less over the whole epitope length. This resulted in a final list of 539 MHCI epitopes, and 1,144 MHCII epitopes. ( *Extended data:* Table 7). We used the datasets obtained for the biogeography analysis (2,061 genome sequences for L1, and 1,021 genome sequences for L3).

For each lineage, we independently assembled a multiple sequence alignment for each epitope. We then translated the sequence to amino acids and used PAUP 4.a ( Wilgenbusch & Swofford, 2003) to reconstruct the replacement history of all polymorphic positions on the rooted phylogenetic trees. We used two maximum parsimony algorithms (ACCTRAN and DELTRAN) and considered only the events reconstructed by both algorithms.

### Analysis of esxH

We considered the first 18 amino acids of *esxH* (MSQIMYNYPAMLGHAGDM), which was by far the epitope with most amino acid replacements in L1. We expanded the analysis of this epitope to the L2 and L4 datasets, so that we could compare the results with L1 and L3. For the PAML analysis, we randomly selected 500 strains from each MTBC lineage. We used the phylogenetic tree reconstructed by RAxML (same settings as above), and the gene alignment to estimate the branch lengths of the tree using the M0 codon model implemented in PAML 4.9e ( Yang, 2007). This step was necessary to obtain a tree with the branch length in expected substitutions per codon. We then fitted two alternative codon models (M1a and M2a) to the trees and alignments. M1a allows ω to be variable across sites, and it assumes two different ω (0 < ω _0_ < 1, and ω _1_ = 1), modeling nearly neutral evolution; M2a assumes one additional ω (ω _2_ > 1) compared to M1a, thus modeling positive selection. We performed a likelihood ratio test between the two models as described in Zhang *et al.* (2005). Templates for the control files of the codeml analyses (M0, M1a, M2a) are available as Supplementary Files in *Extended data*. The codon under positive selection were identified with the Bayes empirical Bayes method ( Yang *et al.*, 2005).

To test whether the derived haplotypes of this epitope were associated with a specific geographic region, we constructed a statistical test similar (but not identical) to PhyC ( Farhat *et al.*, 2013), which is normally used to test for association between a variant and phenotypic drug resistance. For each aminoacid replacement (or combination of aminoacid replacements): we considered the number of independent mutational events that occurred along the tree of L1 (as estimated by PAUP). We then randomly redistributed the same number of replacements on the phylogenetic tree of L1 (based on the branch length; i.e. on longer branches there is a higher probability of a mutational event), and counted the number of strains with a derived state for each geographic region. We built null distributions by repeating this procedure 10,000 times. Under the null hypothesis, mutations occur randomly on the tree, and therefore independently from the geographic region where the tips were sampled. For each region, we ranked the observed value (the number of tips with a derived state) on the corresponding null distribution to obtain empirical p-values. We used the same geographic region used for the biogeography analysis. Additionally, we considered East Africa excluding Madagascar.

R code and input files to perform this test are available as Supplementary Files in *Extended data*.

### Prediction of binding affinity between T cell epitopes and HLA alleles

We considered three HLA loci: HLA-A, HLA-B and HLA-DRB1. We used the allele frequency database ( Gonzalez-Galarza *et al.*, 2020) to identify alleles that are prevalent in East Africa and not in South Asia and Southeast Asia, or the other way around. Because the coverage of the allele frequency database is patchy, we focused on the following countries: Kenya and Zimbabwe as representatives of East Africa; India, Thailand and Taiwan as representatives of South- and Southeast Asia. We identified:

1) Alleles that had a frequency of 10% or more in at least one population in South- and Southeast Asia, but had frequencies lower than 10% in all East African populations.

2) Alleles that had a frequency of 10% or more in at least one population in East Africa, but had frequencies lower than 10% in all populations in South- and Southeast Asia.

3) Alleles that had a frequency of 10% or more in at least one population both in South- and Southeast Asia and in East Africa.

For all these alleles, we performed *in silico* binding prediction with three epitopes: the ancestral epitope at the *esxH* N terminus (MSQIMYNYPAMLGHAGDM), and the two most frequently observed derived epitopes (MSQIMYNYPTMLGHAGDM, and MSQIMYNYPVMLGHAGDM). For HLA-A and HLA-B alleles, we used the NetMHCPan4.1 server ( Reynisson *et al.*, 2020) with standard settings. For HLA-DRB1 alleles, we used the prediction tool of the immune epitope database ( https://www.iedb.org/).

### Genome wide scan for positive selection with PAML

For this analysis, we used the subsets generated for the Mascot analysis. These datasets are representative of the populations of L1 and L3 in their core geographic ranges, and are computationally treatable. We generated sequence alignments for all genes individually, excluding genes in repetitive regions of the genome (see above). Because some genes are deleted in L1 but not in L3, or the other way around, we obtained a slightly different number of gene alignments for the two lineages (L1: 3,623, L3: 3,622). For each gene, we performed a test for positive selection with PAML as described above for *esxH*. We considered as under positive selection all genes, for which the likelihood ratio test resulted in a Bonferroni-corrected p-value < 0.05.

### Drug resistance mutations profiles

We considered 196 mutations conferring resistance to different antibiotics ( Payne *et al.*, 2019). We extracted the respective genomic positions from the vcf file of the 4,968 genomes of the complete curated data set (2,938 L1, 2,030 L3) and assembled them in phylip format. To determine the number of independent mutations, we reconstructed the nucleotide changes on the phylogenetic tree rooted with the L2 strain (SAMEA4441446). To do this, we used the maximum parsimony ACCTRAN and DELTRAN algorithms implemented in PAUP 4.0a ( Wilgenbusch & Swofford, 2003), and considered only the events reconstructed by both algorithms.

## Data availability

### Underlying data

The sequence data generated by this study has been deposited on SRA ( https://www.ncbi.nlm.nih.gov/sra) under the accession number PRJNA670836.

### Extended data

Extended data is available here: https://github.com/fmenardo/MTBC_L1_L3.

DOI: https://doi.org/10.5281/zenodo.4609804 ( Menardo, 2021).

This project contains the following extended data:

- The folder Supplementary_text_figures_tables contains supplementary text, figures and tables.
- Supplementary_file_1.html: PASTML results for L1, compressed tree.
- Supplementary_file_2.html: PASTML results for L1, complete tree.
- Supplementary_file_3.html: PASTML results for L3, compressed tree.
- Supplementary_file_4.html: PASTML results for L3, complete tree.
- The folder Dating_Beast contains the xml files for the dating analyses.
- The folder EsxH_haplotype_association_test contains the code and input files to perform the test of association between different haplotypes and geographic regions.
- The folder EsxH_PAML contains the control files and input files to perform the positive selection analysis on EsxH.
- The folder Mascot contains the xml files for the Mascot analyses.

Extended data is available under a GNU GENERAL PUBLIC LICENSE.

## References

[ref-1] Abel B Tameris M Mansoor N: The novel tuberculosis vaccine, AERAS-402, induces robust and polyfunctional CD4 ^+^ and CD8 ^+^ T cells in adults. *Am J Respir Crit Care Med.* 2010;181(12):1407–1417. 10.1164/rccm.200910-1484OC 20167847 PMC2894413

[ref-2] Allen RB: Indian Ocean transoceanic migration, 16th–19th century. *The Encyclopedia of Global Human Migration.* 2013. 10.1002/9781444351071.wbeghm294

[ref-3] Bekker LG Dintwe O Fiore-Gartland A: A phase 1b randomized study of the safety and immunological responses to vaccination with H4:IC31, H56:IC31, and BCG revaccination in *Mycobacterium tuberculosis*-uninfected adolescents in Cape Town, South Africa. *EClinicalMedicine.* 2020;21:100313. 10.1016/j.eclinm.2020.100313 32382714 PMC7201034

[ref-91] Belisle JT Sonnenberg MG: Isolation of genomic DNA from mycobacteria.In *Mycobacteria protocols.*Humana Press,1998;31–44. 10.1385/0-89603-471-2:31 9921467

[ref-4] Blench R: Evidence for the Austronesian voyages in the Indian Ocean. *The Global Origins and Development of Seafaring.* 2010;239:48. Reference Source

[ref-5] Boivin N Crowther A Helm R: East Africa and Madagascar in the Indian Ocean world. *J World Prehist.* 2013;26(3):213–281. 10.1007/s10963-013-9067-4

[ref-6] Bolger AM Lohse M Usadel B: Trimmomatic: a flexible trimmer for Illumina sequence data. *Bioinformatics.* 2014;30(15):2114–2120. 10.1093/bioinformatics/btu170 24695404 PMC4103590

[ref-7] Bos KI Harkins KM Herbig A: Pre-Columbian mycobacterial genomes reveal seals as a source of New World human tuberculosis. *Nature.* 2014;514(7523):494–497. 10.1038/nature13591 25141181 PMC4550673

[ref-8] Bottai D Frigui W Sayes F: TbD1 deletion as a driver of the evolutionary success of modern epidemic *Mycobacterium tuberculosis* lineages. *Nat Commun.* 2020;11(1):684. 10.1038/s41467-020-14508-5 32019932 PMC7000671

[ref-9] Bouckaert R Vaughan TG Barido-Sottani J: BEAST 2.5: An advanced software platform for Bayesian evolutionary analysis. *PLoS Comput Biol.* 2019;15(4):e1006650. 10.1371/journal.pcbi.1006650 30958812 PMC6472827

[ref-10] Brahmajothi V Pitchappan RM Kakkanaiah VN: Association of pulmonary tuberculosis and HLA in south India. *Tubercle.* 1991;72(2):123–132. 10.1016/0041-3879(91)90039-u 1949215

[ref-11] Brites D Loiseau C Menardo F: A new phylogenetic framework for the animal-adapted *Mycobacterium tuberculosis* complex. *Front Microbiol.* 2018;9:2820. 10.3389/fmicb.2018.02820 30538680 PMC6277475

[ref-14] Brucato N Fernandes V Kusuma P: Evidence of Austronesian Genetic Lineages in East Africa and South Arabia: Complex Dispersal from Madagascar and Southeast Asia. *Genome Biol Evol.* 2019;11(3):748–758. 10.1093/gbe/evz028 30715341 PMC6423374

[ref-13] Brucato N Fernandes V Mazières S: The Comoros Show the Earliest Austronesian Gene Flow into the Swahili Corridor. *Am J Hum Genet.* 2018;102(1):58–68. 10.1016/j.ajhg.2017.11.011 29304377 PMC5777450

[ref-12] Brucato N Kusuma P Cox MP: Malagasy Genetic Ancestry Comes from an Historical Malay Trading Post in Southeast Borneo. *Mol Biol Evol.* 2016;33(9):2396–2400. 10.1093/molbev/msw117 27381999 PMC4989113

[ref-92] Brynildsrud OB Pepperell CS Suffys P: Global expansion of *Mycobacterium tuberculosis* lineage 4 shaped by colonial migration and local adaptation. *Sci Adv.* 2018;4(10):eaat5869. 10.1126/sciadv.aat5869 30345355 PMC6192687

[ref-15] Chihota VN Niehaus A Streicher EM: Geospatial distribution of *Mycobacterium tuberculosis* genotypes in Africa. *PLoS One.* 2018;13(8):e0200632. 10.1371/journal.pone.0200632 30067763 PMC6070189

[ref-16] Coll F McNerney R Guerra-Assuncao JA: A robust SNP barcode for typing *Mycobacterium tuberculosis* complex strains. *Nat Commun.* 2014;5(1):4812. 10.1038/ncomms5812 25176035 PMC4166679

[ref-17] Comas I Chakravartti J Small PM: Human T cell epitopes of Mycobacterium tuberculosis are evolutionarily hyperconserved. * Nat Genet.* 2010;42(6):498–503. 10.1038/ng.590 20495566 PMC2883744

[ref-18] Comas I Coscolla M Luo T: Out-of-Africa migration and Neolithic coexpansion of *Mycobacterium tuberculosis* with modern humans. *Nat Genet* 2013;45(10):1176–1182. 10.1038/ng.2744 23995134 PMC3800747

[ref-19] Conceição EC Refregier G Gomes HM: *Mycobacterium tuberculosis* lineage 1 genetic diversity in Pará, Brazil, suggests common ancestry with east-African isolates potentially linked to historical slave trade. *Infect Genet Evol.* 2019;73:337–341. 10.1016/j.meegid.2019.06.001 31170529

[ref-24] Coscollá M Brites D Menardo F: Phylogenomics of *Mycobacterium africanum* reveals a new lineage and a complex evolutionary history. * Microb Genom.* 2021;7(2). 10.1099/mgen.0.000477 33555243 PMC8208692

[ref-20] Coscolla M Copin R Sutherland J: *M. tuberculosis* T Cell Epitope Analysis Reveals Paucity of Antigenic Variation and Identifies Rare Variable TB Antigens. *Cell Host Microbe.* 2015;18(5):538–548. 10.1016/j.chom.2015.10.008 26607161 PMC4758912

[ref-21] Couvin D Reynaud Y Rastogi N: Two tales: Worldwide distribution of Central Asian (CAS) versus ancestral East-African Indian (EAI) lineages of *Mycobacterium tuberculosis* underlines a remarkable cleavage for phylogeographical, epidemiological and demographical characteristics. *PLoS One.* 2019;14(7):e0219706. 10.1371/journal.pone.0219706 31299060 PMC6625721

[ref-22] Darriba D Taboada GL Doallo R: jModelTest 2: more models, new heuristics and parallel computing. *Nat Methods.* 2012;9(8):772. 10.1038/nmeth.2109 22847109 PMC4594756

[ref-23] De Jong BC Antonio M Gagneux S: *Mycobacterium africanum*--review of an important cause of human tuberculosis in West Africa. *PLoS Negl Trop Dis.* 2010;4(9):e744. 10.1371/journal.pntd.0000744 20927191 PMC2946903

[ref-25] Dou HY Chen YY Kou SC: Prevalence of *Mycobacterium tuberculosis* strain genotypes in Taiwan reveals a close link to ethnic and population migration. *J Formos Med Assoc.* 2015;114(6):484–488. 10.1016/j.jfma.2014.07.006 25542769

[ref-26] Drummond AJ Ho SYW Phillips MJ: Relaxed phylogenetics and dating with confidence. *PLoS Biol.* 2006;4(5):e88. 10.1371/journal.pbio.0040088 16683862 PMC1395354

[ref-27] Duchêne S Holt KE Weill FX: Genome-scale rates of evolutionary change in bacteria. *Microb Genom.* 2016;2(11):e000094. 10.1099/mgen.0.000094 28348834 PMC5320706

[ref-28] Farhat MR Shapiro BJ Kieser KJ: Genomic analysis identifies targets of convergent positive selection in drug-resistant *Mycobacterium tuberculosis*. *Nat Genet.* 2013;45(10):1183–1189. 10.1038/ng.2747 23995135 PMC3887553

[ref-29] Fenner L Egger M Bodmer T: Effect of mutation and genetic background on drug resistance in *Mycobacterium tuberculosis*. *Antimicrob Agents Chemother.* 2012;56(6):3047–3053. 10.1128/AAC.06460-11 22470121 PMC3370767

[ref-30] Gagneux S (Ed.): Strain variation in the Mycobacterium tuberculosis complex: its role in biology, Epidemiology and Control. *Springer.* 2017;1019. 10.1007/978-3-319-64371-7

[ref-31] Gagneux S: Ecology and evolution of *Mycobacterium tuberculosis*. *Nat Rev Microbiol.* 2018;16(4):202–213. 10.1038/nrmicro.2018.8 29456241

[ref-32] Glaziou P: Predicted impact of the COVID-19 pandemic on global tuberculosis deaths in 2020. *medRxiv.* 2020. 10.1101/2020.04.28.20079582

[ref-33] Gonzalez-Galarza FF McCabe A Santos EJMD: Allele frequency net database (AFND) 2020 update: gold-standard data classification, open access genotype data and new query tools. *Nucleic Acids Res.* 2020;48(D1):D783–D788. 10.1093/nar/gkz1029 31722398 PMC7145554

[ref-34] Ho SYW Phillips MJ Cooper A: Time dependency of molecular rate estimates and systematic overestimation of recent divergence times. *Mol Biol Evol.* 2005;22(7):1561–1568. 10.1093/molbev/msi145 15814826

[ref-35] Hoang T Aagaard C Dietrich J: ESAT-6 (EsxA) and TB10.4 (EsxH) based vaccines for pre- and post-exposure tuberculosis vaccination. *PLoS One.* 2013;8(12):e80579. 10.1371/journal.pone.0080579 24349004 PMC3861245

[ref-36] Hogan AB Jewell BL Sherrard-Smith E: Potential impact of the COVID-19 pandemic on HIV, tuberculosis, and malaria in low-income and middle-income countries: a modelling study. *Lancet Glob Health.* 2020;8(9):e1132–e1141. 10.1016/S2214-109X(20)30288-6 32673577 PMC7357988

[ref-37] Holt KE McAdam P Thai PVK: Frequent transmission of the *Mycobacterium tuberculosis* Beijing lineage and positive selection for the EsxW Beijing variant in Vietnam. *Nat Genet.* 2018;50(6):849–856. 10.1038/s41588-018-0117-9 29785015 PMC6143168

[ref-38] Ilghari D Lightbody KL Veverka V: Solution Structure of the *Mycobacterium tuberculosis* EsxG·EsxH Complex functional implications and comparisons with other m. tuberculosis esx family complexes. *J Biol Chem.* 2011;286(34):29993–30002. 10.1074/jbc.M111.248732 21730061 PMC3191040

[ref-39] Ishikawa SA Zhukova A Iwasaki W: A fast likelihood method to reconstruct and visualize ancestral scenarios. *Mol Biol Evol.* 2019;36(9):2069–2085. 10.1093/molbev/msz131 31127303 PMC6735705

[ref-40] Koboldt DC Zhang Q Larson DE: VarScan 2: somatic mutation and copy number alteration discovery in cancer by exome sequencing. *Genome Res.* 2012;22(3):568–576. 10.1101/gr.129684.111 22300766 PMC3290792

[ref-42] Li H Durbin R: Fast and accurate long-read alignment with Burrows-Wheeler transform. *Bioinformatics.* 2010;26(5):589–595. 10.1093/bioinformatics/btp698 20080505 PMC2828108

[ref-41] Li H Handsaker B Wysoker A: The sequence alignment/map format and SAMtools. *Bioinformatics.* 2009;25(16):2078–2079. 10.1093/bioinformatics/btp352 19505943 PMC2723002

[ref-96] Loiseau C Menardo F Aseffa A: An African origin for *Mycobacterium bovis*. *Evol Med Public Health.* 2020;2020(1):49–59. 10.1093/emph/eoaa005 32211193 PMC7081938

[ref-44] McKenna A Hanna M Banks E: The Genome Analysis Toolkit: a MapReduce framework for analyzing next-generation DNA sequencing data. * Genome Res.* 2010;20(9):1297–1303. 10.1101/gr.107524.110 20644199 PMC2928508

[ref-45] McQuaid CF McCreesh N Read JM: The potential impact of COVID-19-related disruption on tuberculosis burden. *Eur Respir J.* 2020;56(2):2001718. 10.1183/13993003.01718-2020 32513784 PMC7278504

[ref-46] Mehra A Zahra A Thompson V: **Mycobacterium tuberculosis** type VII secreted effector EsxH targets host ESCRT to impair trafficking. *PLoS Pathog.* 2013;9(10): e1003734. 10.1371/journal.ppat.1003734 24204276 PMC3814348

[ref-43] Menardo F: fmenardo/MTBC_L1_L3: MTBC L1 L3 (Version v2). *Zenodo.* 2021. 10.5281/zenodo.4435760

[ref-48] Menardo F Duchêne S Brites D: The molecular clock of *Mycobacterium tuberculosis*. *PLoS Pathog.* 2019;15(9): e1008067. 10.1371/journal.ppat.1008067 31513651 PMC6759198

[ref-47] Menardo F Loiseau C Brites D: Treemmer: a tool to reduce large phylogenetic datasets with minimal loss of diversity. *BMC Bioinformatics.* 2018;19(1):164. 10.1186/s12859-018-2164-8 29716518 PMC5930393

[ref-49] Mittal E Skowyra ML Uwase G: *Mycobacterium tuberculosis* type VII secretion system effectors differentially impact the ESCRT endomembrane damage response. *mBio.* 2018;9(6): e01765–18. 10.1128/mBio.01765-18 30482832 PMC6282207

[ref-50] Müller NF Rasmussen D Stadler T: MASCOT: parameter and state inference under the marginal structured coalescent approximation. *Bioinformatics.* 2018;34(22):3843–3848. 10.1093/bioinformatics/bty406 29790921 PMC6223361

[ref-95] Mulholland CV Shockey AC Aung HL: Dispersal of *Mycobacterium tuberculosis* driven by historical European trade in the South Pacific. *Front Microbiol.* 2019;10:2778. 10.3389/fmicb.2019.02778 31921003 PMC6915100

[ref-51] Nemes E Geldenhuys H Rozot V: Prevention of *M. tuberculosis* infection with H4:IC31 vaccine or BCG revaccination. *N Engl J Med.* 2018;379(2):138–149. 10.1056/NEJMoa1714021 29996082 PMC5937161

[ref-94] Netikul T Palittapongarnpim P Thawornwattana Y: Estimation of the global burden of *Mycobacterium tuberculosis* lineage 1. *Infect Genet Evol.* 2021;91:104802. 10.1016/j.meegid.2021.104802 33684570

[ref-52] Ngabonziza JCS Loiseau C Marceau M: A sister lineage of the *Mycobacterium tuberculosis* complex discovered in the African Great Lakes region. *Nat Commun.* 2020;11(1):2917. 10.1038/s41467-020-16626-6 32518235 PMC7283319

[ref-53] O'Neill MB Shockey A Zarley A: Lineage specific histories of *Mycobacterium tuberculosis* dispersal in Africa and Eurasia. *Mol Ecol.* 2019;28(13):3241–3256. 10.1111/mec.15120 31066139 PMC6660993

[ref-60] Qu HQ Fisher-Hoch SP McCormick JB: Knowledge gaining by human genetic studies on tuberculosis susceptibility. *J Hum Genet.* 2011;56(3):177–182. 10.1038/jhg.2010.164 21179108 PMC3169186

[ref-54] Payne JL Menardo F Trauner A: Transition bias influences the evolution of antibiotic resistance in *Mycobacterium tuberculosis*. *PLoS Biol.* 2019;17(5): e3000265. 10.1371/journal.pbio.3000265 31083647 PMC6532934

[ref-55] Pepperell CS Casto AM Kitchen A: The role of selection in shaping diversity of natural *M. tuberculosis* populations. *PLoS Pathog.* 2013;9(8): e1003543. 10.1371/journal.ppat.1003543 23966858 PMC3744410

[ref-56] Peters JS Ismail N Dippenaar A: Genetic Diversity in **Mycobacterium tuberculosis** Clinical Isolates and Resulting Outcomes of Tuberculosis Infection and Disease. *Annu Rev Genet.* 2020;54:511–537. 10.1146/annurev-genet-022820-085940 32926793 PMC12646202

[ref-57] Pierron D Razafindrazaka H Pagani L: Genome-wide evidence of Austronesian-Bantu admixture and cultural reversion in a hunter-gatherer group of Madagascar. *Proc Natl Acad Sci U S A.* 2014;111(3):936–941. 10.1073/pnas.1321860111 24395773 PMC3903192

[ref-58] Portal-Celhay C Tufariello JM Srivastava S: *Mycobacterium tuberculosis* EsxH inhibits ESCRT-dependent CD4 ^+^ T-cell activation. *Nat Microbiol.* 2016;2(2):16232. 10.1038/nmicrobiol.2016.232 27918526 PMC5453184

[ref-59] Price MN Dehal PS Arkin AP: FastTree 2--approximately maximum-likelihood trees for large alignments. *PLoS One.* 2010;5(3):e9490. 10.1371/journal.pone.0009490 20224823 PMC2835736

[ref-61] Rabahi MF Conceição EC de Paiva LO: Characterization of *Mycobacterium tuberculosis* var. *africanum* isolated from a patient with pulmonary tuberculosis in Brazil. *Infect Genet Evol.* 2020;85:104550. 10.1016/j.meegid.2020.104550 32920193

[ref-62] Radošević K Wieland CW Rodriguez A: Protective immune responses to a recombinant adenovirus type 35 tuberculosis vaccine in two mouse strains: CD4 and CD8 T-cell epitope mapping and role of gamma interferon. *Infect Immun.* 2007;75(8):4105–4115. 10.1128/IAI.00004-07 17526747 PMC1951991

[ref-63] Rambaut A Drummond AJ Xie D: Posterior summarization in Bayesian phylogenetics using Tracer 1.7. *Syst Biol.* 2018;67(5):901–904. 10.1093/sysbio/syy032 29718447 PMC6101584

[ref-64] Reynisson B Alvarez B Paul S: NetMHCpan-4.1 and NetMHCIIpan-4.0: improved predictions of MHC antigen presentation by concurrent motif deconvolution and integration of MS MHC eluted ligand data. *Nucleic Acids Res.* 2020;48(W1):W449–W454. 10.1093/nar/gkaa379 32406916 PMC7319546

[ref-65] Russel PM Brewer BJ Klaere S: Model selection and parameter inference in phylogenetics using Nested Sampling. *Syst Biol.* 2019;68(2):219–233. 10.1093/sysbio/syy050 29961836

[ref-93] Rutaihwa LK Menardo F Stucki D: Multiple introductions of *Mycobacterium tuberculosis* lineage 2–Beijing into Africa over centuries. *Front Ecol Evol.* 2019;7:112. 10.3389/fevo.2019.00112

[ref-66] Salie M van der Merwe L Möller M: Associations between human leukocyte antigen class I variants and the *Mycobacterium tuberculosis* subtypes causing disease. *J Infect Dis.* 2014;209(2):216–223. 10.1093/infdis/jit443 23945374 PMC3873786

[ref-67] Saunders MJ Evans CA: COVID-19, tuberculosis and poverty: preventing a perfect storm. *Eur Respir J.* 2020;56(1):2001348. 10.1183/13993003.01348-2020 32444399 PMC7243392

[ref-68] Smittipat N Miyahara R Juthayothin T: Indo-Oceanic *Mycobacterium tuberculosis* strains from Thailand associated with higher mortality. *Int J Tuberc Lung Dis.* 2019;23(9):972–979. 10.5588/ijtld.18.0710 31615603

[ref-69] Stamatakis A: RAxML version 8: a tool for phylogenetic analysis and post-analysis of large phylogenies. *Bioinformatics.* 2014;30(9):1312–1313. 10.1093/bioinformatics/btu033 24451623 PMC3998144

[ref-70] Steiner A Stucki D Coscolla M: KvarQ: targeted and direct variant calling from fastq reads of bacterial genomes. *BMC Genomics.* 2014;15(1):881. 10.1186/1471-2164-15-881 25297886 PMC4197298

[ref-71] Sutiwisesak R Hicks ND Boyce S: A natural polymorphism of *Mycobacterium tuberculosis* in the *esxH* gene disrupts immunodomination by the TB10.4-specific CD8 T cell response. *PLoS Pathog.* 2020;16(10):e1009000. 10.1371/journal.ppat.1009000 33075106 PMC7597557

[ref-72] Sveinbjornsson G Gudbjartsson DF Halldorsson BV: *HLA* class II sequence variants influence tuberculosis risk in populations of European ancestry. *Nat Genet.* 2016;48(3):318–322. 10.1038/ng.3498 26829749 PMC5081101

[ref-73] To TH Jung M Lycett S: Fast dating using least-squares criteria and algorithms. *Syst Biol.* 2016;65(1):82–97. 10.1093/sysbio/syv068 26424727 PMC4678253

[ref-74] Vejbaesya S Chierakul N Luangtrakool K: Associations of HLA class II alleles with pulmonary tuberculosis in Thais. *Eur J Immunogenet.* 2002;29(5):431–434. 10.1046/j.1365-2370.2002.00352.x 12358854

[ref-75] White Z Painter J Douglas P: Immigrant arrival and tuberculosis among large immigrant- and refugee-receiving countries, 2005–2009. *Tuberc Res Treat.* 2017;2017:8567893. 10.1155/2017/8567893 28424748 PMC5382300

[ref-76] Wiens KE Woyczynski LP Ledesma JR: Global variation in bacterial strains that cause tuberculosis disease: a systematic review and meta-analysis. *BMC Med.* 2018;16(1):196. 10.1186/s12916-018-1180-x 30373589 PMC6206891

[ref-77] Wilgenbusch JC Swofford D: Inferring evolutionary trees with PAUP. *Curr Protoc Bioinformatics.* 2003; (1):6–4. 10.1002/0471250953.bi0604s00 18428704

[ref-78] Woodworth JS Wu Y Behar SM: *Mycobacterium tuberculosis*-specific CD8 ^+^ T cells require perforin to kill target cells and provide protection *in vivo*. *J Immunol.* 2008;181(12):8595–8603. 10.4049/jimmunol.181.12.8595 19050279 PMC3133658

[ref-79] World Health Organization: Global Tuberculosis Report.2020. Reference Source

[ref-81] Yang Z: PAML 4: phylogenetic analysis by maximum likelihood. *Mol Biol Evol.* 2007;24(8):1586–1591. 10.1093/molbev/msm088 17483113

[ref-80] Yang Z Wong WS Nielsen R: Bayes empirical Bayes inference of amino acid sites under positive selection. *Mol Biol Evol.* 2005;22(4):1107–1118. 10.1093/molbev/msi097 15689528

[ref-82] Yuliwulandari R Sachrowardi Q Nakajima H: Association of HLA-A, -B, and -DRB1 with pulmonary tuberculosis in western Javanese Indonesia. *Hum Immunol.* 2010;71(7):697–701. 10.1016/j.humimm.2010.04.005 20438789

[ref-83] Zhang J Nielsen R Yang Z: Evaluation of an improved branch-site likelihood method for detecting positive selection at the molecular level. *Mol Biol Evol.* 2005;22(12):2472–2479. 10.1093/molbev/msi237 16107592

