# Supplementary material for: Local adaptation in populations of Mycobacterium tuberculosis endemic to the Indian Ocean Rim
Source: F1000Res. 2021 Mar 29;10:60. Originally published 2021 Feb 1. [Version 2] doi: 10.12688/f1000research.28318.2 (PMC7921886; doi:10.12688/f1000research.28318.2)
Supplement: Peer review history

# Peer review: Reviewer response for version 1

**DOI:** 10.5256/f1000research.31323.r79710

**Published:** 2021-03-15

**Authors:** Ola B. Brynildsrud

**Affiliations:** 1 Division of Infectious Disease Control and Environmental Health, Norwegian Institute of Public Health, Oslo, Norway

In the paper "Local adaptation in populations of Mycobacterium tuberculosis endemic to the Indian Ocean Rim", Menardo and colleagues explore diversity, phylogeographic relationships and patterns of adaptation in TB lineages 1 and 3, both of which are distributed mainly around the Indian Ocean. The paper demonstrates a strongly asymmetric trend of South Asian spread to other regions. Further, it describes a search for positive selection throughout these genomes, and identify one gene in particular, exsH, as being a target for T-cell dependent selection. The authors additionally calibrates molecular clocks for both L1 and L3 and use this to date certain events in the tree.

 The methodology is solid and well suited to answer the questions posed. I found the paper to be well written and helpful in shedding light on important questions related to the evolution of L1 and L3. I have only a few minor discussion points.

 Now, the clock analyses are not heavily emphasized, but I thought it might be a potential flaw that the calibration rests on the assumption that the foundation of a single sub-lineage in West Africa could not have happened prior to the establishment of Portuguese sailing routes around the tip of Africa. While I'm certainly not an expert on historic migrations or anthropology, I would assume there must be many possible alternative scenarios. (For example, pilgrimages across the Sahara are known to have happened much prior to this). As far as I can tell, this calibration is the major driving force for the difference in L1 TMRCA from O'Neill. It would be interesting to know how the degree to which this calibration affects the TMRCA and clock rate. On a somewhat related note, this article fails to cite some key references on the phylogeography of TB.

 In one section the authors evaluate association between esxH haplotypes and the geographic region of origin. They calculate this association using a home-made statistical test based on phyC. I could not gather from the description exactly what they measure. It is a simulation-based test that measures "how many strains from each region resulted to have a derived state according to the simulation". Is there a test statistic being ranked here? Is it single-region vs the rest or is the statistic a composite measure of multiple regions? How is the simulation for the null distribution set up? E.g. is it a forward-in-time simulation using a specified mutation rate or a permutation or something else? I see that the code for running this test is posted on github - However, I still think a few more details in the actual paper is in order for the biologists that don't read R. Speaking of this test, It is a bit unconvential for Table 1 to highlight all results with P<0.1, especially since a large number of associations are being tested here. I would not consider p<0.1 to be particularly strong evidence of association.

 I'd like to commend the authors for making so much of their data publicly available. Datasets are in immediately-usable formats which makes it simple to download all the raw data, and as mentioned code is also readily available. 

 Very minor:

- HLA abbreviation never explained in manuscript.
- Tip date randomisation performed, but the results of this test is never stated.
- Why is PRJNA630228 posted? As far as I can tell it doesnt contain any SRA data, and the other accession contains data for all 767 strains.

Is the work clearly and accurately presented and does it cite the current literature?

Yes

If applicable, is the statistical analysis and its interpretation appropriate?

Yes

Are all the source data underlying the results available to ensure full reproducibility?

Yes

Is the study design appropriate and is the work technically sound?

Yes

Are the conclusions drawn adequately supported by the results?

Yes

Are sufficient details of methods and analysis provided to allow replication by others?

Yes

Reviewer Expertise:

Bioinformatics, microbial genomics

I confirm that I have read this submission and believe that I have an appropriate level of expertise to confirm that it is of an acceptable scientific standard.

# Response

**Published:** 2021-03-19

**Authors:** Fabrizio Menardo

**Affiliations:** Swiss Tropical and Public Health Institute, Switzerland

Thank you very much for your comments, and for reviewing our manuscript. We address all your points below. We prepared a revised version of the manuscript based on the feedback of all reviewers.

**Regarding the clock analysis of L1:**

 We performed a tip dating analysis but found no temporal signal for L1. Therefore we used the clock rate estimate resulted from a previous study (Menardo *et al.* 2019) where we analyzed a different L1 dataset and found a good temporal signal and a clock rate of ~1.4x10 ^-7^. So far Menardo *et al.* 2019 is the only published study that estimated the clock rate for a L1 dataset.

 In a second step we tested whether this clock rate and two alternative values, 5x10 ^-8 ^and 1.2x10 ^-7,^, (the lowest one was used by O’Neill *et al. *2019) were compatible with the hypothesis of a West African introduction of L1.1.1 posterior to the Portuguese circumnavigation of Africa. We found that only the largest clock rate was compatible with this hypothesis.

 We are quite explicit in the manuscript about the limitation of this analysis, and how it should be interpreted in comparison to the results of O’Neill *et al.* 2019: “This discrepancy is due to different assumptions about the clock rates of L1, but none of the two hypotheses can be excluded with the available data”.

 Finally, it is possible that the West African L1.1.1 clade did not originate through a direct migration from South East Asia. However, a series of short range migrations seems less likely, as we did not find strains belonging to L1.1.1 in South and Central Asia, or in East Africa.

 As we write in the manuscript, the dating analyses for MTB in general, and for this L1 dataset in particular, have limitations. Here we discussed what we consider the most likely scenario given the data, we did not exclude alternative scenarios.

 We added a section in the discussion comparing our findings with the biogeography of other MTBC lineages, and referencing the relevant studies.

 We revised and expanded the Methods section regarding the phylogenetic test of association to provide more details. To answer the specific questions: the statistic ranked is the number of tips with a derived state; the test is performed for each region individually; the simulation does not use a mutation rate, but it distributes randomly a fixed number of mutations on the tree (the same number inferred from the observed data).

 We also removed special fonts from Table 1, now all p-values have standard formatting.

**Minor points:**

 We now write HLA in full at the first occurrence.

 The results of the DRT are reported in the supplementary Information (now amended to enhance clarity), in Sup. Figs 8 and 9 and in Sup. Table 3. In the main text we refer more generally to the lack of temporal signal (or to a good temporal signal), as this reflects the results of the DRT, but also of the Beast analyses.

 We now refer only to PRJNA670836, which contains data for all the newly sequenced strains. Thanks.

# Peer review: Reviewer response for version 1

**DOI:** 10.5256/f1000research.31323.r79237

**Published:** 2021-03-01

**Authors:** Miguel Viveiros, João Perdigão

**Affiliations:** 1 Global Health and Tropical Medicine, GHTM, Institute of Hygiene and Tropical Medicine, IHMT, NOVA University Lisbon, Lisbon, Portugal; 2 Research Institute for Medicines (iMed.ULisboa), Faculty of Pharmacy, University of Lisbon, Lisbon, Portugal

This study depicts a very interesting and relevant account on the evolutionary history and phylogeography of *Mycobacterium tuberculosis* of Lineages 1 and 3. Historically, and while forming an important part of the *M. tuberculosis* species diversity, the study of both lineages have been neglected in comparison to Lineages 2 and 4. The authors of this study assembled a large genomic dataset encompassing 2938 and 2030 L1 and L3 isolates, respectively, and by implementing distinct molecular evolutionary algorithms were able to put forward a scenario for the dissemination of these strains. A South Asian origin is proposed for both clades from where these strains appeared to have radiated via migration-driven dispersion towards East Africa (L3) and other regions (L1). The weakest aspect of the work is the lack of phylogeographic and historical context of L1 and L3 versus L2 and L4 dissemination, especially L4 in South-America and L2 in Asia in the context of the migratory waves.

 The authors used PASTML and Mascot to conduct the phylogeographic analyses, revealing the migratory pattern of these lineages from South Asia revealing the origins of these two lineages. Several genes involved in virulence and drug resistance were shown to be under positive selection in L1 and L3 strains with important adaptative consequences. Importantly, *esxH* derived haplotypes were not randomly distributed through the geographic range of L1 and the finding of positive selection acting on vaccine candidates, such as this, is important and deserves attention.

 The manuscript is very well written, easy to read and understand and the methods are robust and well applied and described. Important to note the absence of a few important references to other groups work on the third wave migration of migrations in the 16 ^th^ century that impacted the dissemination of L1 in Africa and other regions.

**Some comments that can be useful for discussion:**

 The analysis pertaining the introduction of L1 to South America is based on a more modest number of isolates (n=77), all but one from Brazil. This limits the robusteness of the conclusions for this region. It is possible that the evolutionary history of L1 in South America is far more complex than the one outlined in the article and there may be missing links. For example, EAI (L1) strains have been detected in several South and Central American countries (Mexico, Peru, Panama, Colombia, etc.) and besides slave trade, the Spain-operated Manila-Acapulco Galleon trade route (est. ca. 1568) connected Southeast Asia to Central and South America.

 Regarding the L1.1.1 sub-lineage, what precludes these strains to have been introduced to West Africa in a more recent period other than the XVI or the XVII century? As far as we can tell, this pertains a group of 10 isolates scattered over five countries, but what is the degree of divergence between these isolates? Eventually, can this also be linked with the indentured labor promoted by the British Empire in the XIX century?

 On the epitope analysis, upon downloading epitopes from IEDB did the authors include only epitopes with positive assays and was there a threshold for including only epitopes validated by independent assays (more reliable)? Also, how did overlapping epitopes been handled?

Is the work clearly and accurately presented and does it cite the current literature?

Yes

If applicable, is the statistical analysis and its interpretation appropriate?

Yes

Are all the source data underlying the results available to ensure full reproducibility?

Yes

Is the study design appropriate and is the work technically sound?

Yes

Are the conclusions drawn adequately supported by the results?

Yes

Are sufficient details of methods and analysis provided to allow replication by others?

Yes

Reviewer Expertise:

Mycobacteriology, Tuberculosis, Laboratory Diagnosis and Molecular Epidemiology.

We confirm that we have read this submission and believe that we have an appropriate level of expertise to confirm that it is of an acceptable scientific standard.

# Response

**Published:** 2021-03-19

**Authors:** Fabrizio Menardo

**Affiliations:** Swiss Tropical and Public Health Institute, Switzerland

Thank you very much for reviewing and commenting our manuscript. We prepared a revised version of the manuscript based on the feedback of all reviewers.

 Regarding the comparison with other lineages: in this manuscript we focused on L1 and L3 because they are understudied. We now revised the text and added a section comparing our findings with the biogeography of other MTBC lineages, and referencing the relevant studies. Additionally, it is true that the evolutionary history of L1 in South America could be more complex, and we might fail to capture it because we have samples from a single country (Brazil). We now explicitly write this.

 Regarding the L1.1.1 sub-lineage: in the manuscript we write that the 16 ^th^ century is the earliest possible time for the introduction of L1 in West Africa (assuming that the migration could not have happened before the circumnavigation of Africa by Portuguese), we do not exclude the possibility of a later introduction. However, this group of 11 L1 strains comprising the West African clade has a relatively old MRCA. To be compatible with an introduction in the 19 ^th^ century, the molecular clock rate would need to be about 3x10 ^-7^ (this is a back of the envelope calculation, and not the result of a proper analysis). This would be a fast rate for MTB (Menardo et al. 2019), and we find this hypothesis unlikely. Nevertheless, because of the lack of a temporal signal we cannot exclude a more recent introduction of L1 in West Africa.

 Finally, in the epitope analysis we downloaded all T cell epitopes, to be as inclusive as possible. Overlapping epitopes were analyzed independently. We added this information to the manuscript.

# Peer review: Reviewer response for version 1

**DOI:** 10.5256/f1000research.31323.r78953

**Published:** 2021-02-17

**Authors:** Tanvi Honap

**Affiliations:** 1 University of Oklahoma, Norman, OK, USA

The aim of this study was to conduct a phylogeographic analysis of *Mycobacterium tuberculosis* complex (MTBC) strains belonging to Lineages 1 and 3, which are the most prevalent lineages in South Asian countries with a high TB burden, such as India. The authors compiled an excellent dataset of nearly 5,000 MTBC genomes from these two lineages, comprising publicly available as well as newly-sequenced MTBC strains. Their phylogenomic analyses showed that L1 comprises a further five distinct sublineages, whereas L3 does not contain distinct sublineages. The authors used two different methods (PASTML and Mascot) for conducting phylogeographic analyses, with results from both methods suggesting an interesting asymmetric pattern of migration: these lineages originated in and migrated from South Asia to other regions, with very little back-migration into South Asia. This underscores the important role South Asia has played in the dispersal of these lineages. The authors found several genes involved in virulence and antibiotic resistance to be under positive selection in L1 and L3 strains. Lastly, the authors also found evidence of local adaptation at the esxH epitope in L1 strains from South and Southeast Asia, which requires further study as this epitope is a part of several vaccine candidates. 

 Overall, I found this to be a well-designed and thorough study, and a very well-written manuscript. I have no major concerns and only a few minor suggestions for aesthetic improvement:

- The Methods section titled " *Strain cultures, DNA extraction and genome sequencing*" is missing citations for the methods used (eg. CTAB extraction method).
- Figures 1A and 2A heatmaps would look better in color than grayscale. 
- The Supplementary Information would benefit from a thorough proofreading for grammatical and typological errors. 

Is the work clearly and accurately presented and does it cite the current literature?

Yes

If applicable, is the statistical analysis and its interpretation appropriate?

Yes

Are all the source data underlying the results available to ensure full reproducibility?

Yes

Is the study design appropriate and is the work technically sound?

Yes

Are the conclusions drawn adequately supported by the results?

Yes

Are sufficient details of methods and analysis provided to allow replication by others?

Yes

Reviewer Expertise:

Pathogen genomics

I confirm that I have read this submission and believe that I have an appropriate level of expertise to confirm that it is of an acceptable scientific standard.

# Response

**Published:** 2021-03-19

**Authors:** Fabrizio Menardo

**Affiliations:** Swiss Tropical and Public Health Institute, Switzerland

Thank you very much for your comments, and for reviewing our manuscript. We prepared a revised version of the manuscript based on the feedback of all reviewers.

 We added the missing reference for the DNA extraction method, and we revised the supplementary material for errors and typos, thanks.

 Regarding Figures 1A and 2A: in both figures the other three panels have the same color code, where different colors correspond to different world regions. We decided to use a gray scale to avoid potential confusion of having two different color codes in the same figure. We did not modify the figures.
